# Direct SpoIIQ-SpoIIIAH interaction is dispensable for sporulation in *Bacillus subtilis*

**DOI:** 10.1016/j.jbc.2025.110934

**Published:** 2025-11-12

**Authors:** Katarína Muchová, Andrea Vetráková, James A. Brannigan, Sonam Sidhu, Jana Júdová, Zuzana Chromiková, Anthony J. Wilkinson, Imrich Barák

**Affiliations:** 1 Department of Microbial Genetics, Institute of Molecular Biology, Slovak Academy of Sciences, Bratislava, Slovakia; 2 York Structural Biology Laboratory, York Biomedical Research Institute and Department of Chemistry, University of York, York, United Kingdom

**Keywords:** *Bacillus subtilis*, sporulation, protein–protein interaction, SpoIIQ, SpoIIIAH, cell engulfment, sigma factors

## Abstract

*Bacillus subtilis* sporulation involves a fascinating phagocytic process in which the mother cell engulfs the forespore, internalizing the latter as a cell-within-a-cell. Peptidoglycan remodelling machinery, along with the SpoIIIAA-AH:SpoIIQ complex, are crucial to this process. The forespore protein SpoIIQ and the mother cell protein SpoIIIAH, which localize to opposite sides of the sporulation septum, are indispensable for sporulation. These proteins interact through their extracytoplasmic domains across the intermembrane space and are proposed to contribute to an intercellular zipper and/or a channel connecting the forespore and the mother cell. Here, we show using (1) site-directed mutagenesis of SpoIIQ, (2) *in vivo* and *in vitro* interaction and localization studies, and (3) σ^G^ activation and sporulation assays that spores are formed efficiently from cells in which direct interaction between SpoIIIAH and SpoIIQ (H-Q) is disrupted. We propose that the H-Q interaction is dispensable for sporulation and that the essential function of SpoIIQ is in recruitment of other components to the septum/engulfment complex such as SpoIIE, GerM and/or the other SpoIIIA proteins.

Sporulation in the rod-shaped bacterium *Bacillus subtilis* is a starvation response. The first clear morphological feature of sporulation is the formation of an asymmetrically positioned sporulation septum, which is a foundation for establishing differential gene expression in the smaller forespore and the larger mother cell (1). Each compartment inherits an identical copy of the chromosome of the parent cell but the pattern of gene expression, orchestrated by cell-type specific RNA polymerase sigma factors (σ^F^, σ^E^, σ^G^, and σ^K^) differs and the cells have different fates (2, 3). In a phagocytosis event, the mother cell engulfs the forespore, internalizing the latter as a cell-within-a-cell surrounded by a double membrane; an inner membrane originating from the forespore membrane, and an outer membrane derived from the engulfing mother cell (Fig. 1*A*) (4). This engulfment process requires peptidoglycan remodelling: peptidoglycan degradation carried out by a complex of three mother cell proteins SpoIID, SpoIIP, and SpoIIM (the DPM complex) (5) and peptidoglycan synthesis carried out by forespore peptidoglycan-synthesizing enzymes (6, 7). The second module contributing to engulfment consists of the mother cell membrane protein SpoIIIAH, and the forespore membrane protein SpoIIQ, which form intercellular interactions that facilitate membrane migration. Both proteins are important for sporulation, as they are required for the activation of the late forespore sigma factor σ^G^ (8, 9) and the maintenance of the structural integrity of the forespore after engulfment (10).

**Figure 1:**
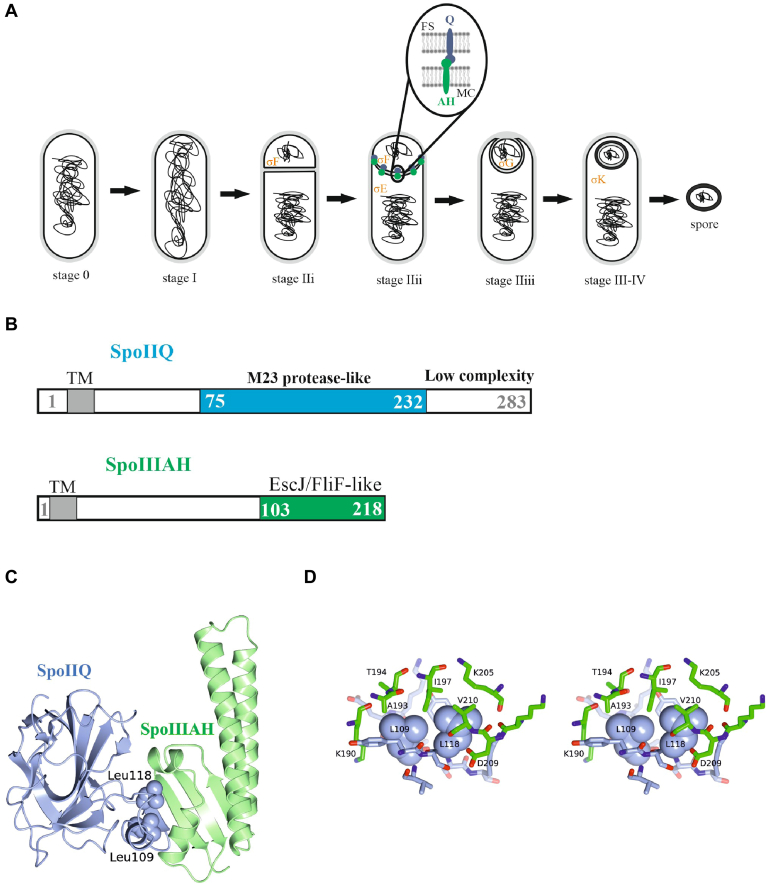
**The process of engulfment and model of the SpoIIQ-SpoIIIAH complex**. *A*, schematic representation of the sporulation program in *Bacillus subtilis* showing compartment specific gene transcription governed by σ factors and the morphological process of engulfment. Stage I represents the formation of the DNA axial filament. Stage II marks the formation of the polar septum, which divides the cell into a larger mother cell (MC) and a smaller forespore (FS). Stage II consists of three substages to mark cells with flat sporulation septa (stage IIi), curved septa (stage IIii) and engulfing septa (stage IIiii). Stage III-IV follows the completion of engulfment of the forespore by the mother cell. Sporulation is completed with the release of the mature spore into the environment. The insert at stage IIii depicts SpoIIQ and SpoIIIAH interacting across the intercellular space. *B*, organization of SpoIIQ and SpoIIIAH proteins. SpoIIQ and SpoIIIAH each have a single N-terminal membrane-spanning segment (TM), SpoIIQ has an extracytoplasmic M23 protease-like domain (residues 75–232) and SpoIIIAH has an EscJ/FliF-like domain (residues 103–218). *C*, structure of the SpoIIQ–SpoIIIAH complex. Ribbon diagram of the complex formed between the extracytoplasmic fragments of SpoIIQ (*blue*) and SpoIIIAH (*green*) PDB: 3TUF. *D*, Stereo detail of the interface. The side chains of residues forming significant intermolecular contacts are shown as cylinders, Leu109 and Leu118 are shown as spheres, the carbon atoms of SpoIIQ are coloured *blue* and those of SpoIIIAH are coloured *green*.

SpoIIQ is a forespore protein whose gene expression is controlled by σ^F^ (11) while SpoIIIAH is a mother cell protein encoded by the last gene of *spoIIIA* operon whose coding sequence is transcribed by RNA polymerase containing σ^E^ (12). Many of the SpoIIIA proteins are related to components of specialized bacterial secretion systems: SpoIIIAA resembles the ATPases of type II and type IV secretion systems; its activity is necessary for σ^G^ activation. SpoIIIAB is also similar to proteins found in Type II and Type IV secretion systems while SpoIIIAE is related to the membrane spanning components of ABC-type permeases involved in type I secretion systems. SpoIIIAF and SpoIIIAH share similarity with proteins found in type III secretion systems (10). The effects of deletion of *spoIIIAH* are less severe than deletion of any of the other seven genes of the *spoIIIA* operon (*spoIIIAA-AG*). The sporulation efficiency of a *ΔspoIIIAH* deletion mutant is 5% of that of wild-type *B*. *subtilis* while the sporulation efficiencies of the deletion mutants *ΔspoIIIAA*, *ΔspoIIIAB*, *ΔspoIIIA*E, *ΔspoIIIAG* and the double mutant *ΔspoIIIACD* are lowered to between 0.003% and 0.001% (10). Deletion of *spoIIQ* decreases the formation of heat-resistant spores by six orders of magnitude with cells blocked at a late stage in engulfment and failing to achieve sporulation stage III (11). Earlier fluorescence microscopy results led to the proposal of an H-Q zipper in which SpoIIIAH and SpoIIQ interact across the intermembrane space, tracking the migration of the engulfing mother cell membrane and stabilizing the double membrane structure (13). Moreover, in protoplasts, devoid of peptidoglycan, SpoIIIAH and SpoIIQ can support the completion of engulfment (14). Sequence analysis suggests that SpoIIIAH and SpoIIQ each have a single N-terminal membrane-spanning segment (Fig. 1*B*). Later, it was recognized that SpoIIQ has an extracytoplasmic M23 metalloprotease-like domain (LytM domain) (15). These domains have been associated with peptidoglycan remodeling. However, SpoIIQ is unlikely to be a protease as it lacks a key residue that would coordinate a catalytic zinc atom. SpoIIIAH possesses a domain associated with proteins found in flagella and type III secretion systems. These proteins form circular assemblies suggesting the existence of an intercellular channel formed by SpoIIIAA-AH and SpoIIQ (16, 17, 18, 19, 20, 21). Structural studies of extracytoplasmic fragments of both SpoIIIAH and SpoIIQ demonstrated that they formed a stable 1:1 complex with a K_d_ of 1 μM (22, 23). Two crystal structures of the H-Q heterodimeric complex reveal how the two proteins, tethered in their respective membranes, might form specific interactions across the intermembrane space. These structures provide structural insight into the nature of the zipper. However, it was not clear how these components form larger functional assemblies. Models of H-Q circular assemblies were built comprising 12-, 15-, and 18-membered rings. How SpoIIQ and SpoIIIAH interact with the remaining SpoIIIA components and how they attach to their respective membranes is not yet understood (22, 23).

Engulfment of the forespore is a complex process in which interaction of SpoIIIAH and SpoIIQ proteins appears to play an important role. In this work, by introducing mutations into SpoIIQ at sites that contribute to the H-Q interface, we investigated the effects of perturbing the H-Q interaction on protein localization, σ^G^ activation, and sporulation efficiency. Our findings indicate that H-Q interaction is dispensable for sporulation and that alternate mechanisms exist to ensure effective sporulation in its absence.

## Results

### Selection of SpoIIQ mutations at the SpoIIIAH—SpoIIQ interface

The H-Q interface is formed by protrusions from each subunit which come together to form an extended intermolecular β-sheet augmented by the close packing of helix α1 of SpoIIQ with helix α3 and a second short helical element of SpoIIIAH (Fig. 1*C*). The SpoIIIAH binding residues of SpoIIQ are primarily contained in the segment Tyr96-Lys120 (Val212 being the only other significant contributor). Meanwhile, the SpoIIQ binding residues of SpoIIIAH are contained in the segment His188-Phe214. The intermolecular main chain-main chain interactions where the β-strands come together feature residues 114 to 116 of SpoIIQ and 212 to 214 of SpoIIIAH (22, 23).

Prominent in the interface are two leucine residues of SpoIIQ, Leu109 and Leu118 whose side-chains project towards SpoIIIAH and become wholly buried in the complex (Fig. 1, *C* and *D*). They bury 46 Å^2^ and 79 Å^2^, respectively, of their accessible surface area in the interface. They form intermolecular interactions with the aliphatic and/or main chain portions of Lys190, Ala193, Thr194, Ile197, Lys205, Lys208, Asp209, and Val210 of SpoIIIAH. These leucines were independently mutated to Ala, Phe, and Glu. Ala is considered a good probe of the contribution a side chain makes to structure and function; Phe is slightly bulkier and may be considered a conservative change while Glu would be considered disruptive in the context of a buried hydrophobic side chain. We also constructed a Glu109-Glu118 double mutant.

### Bacterial two-hybrid analysis revealed differences in interaction characteristics of SpoIIQ mutant proteins

**Figure 2:**
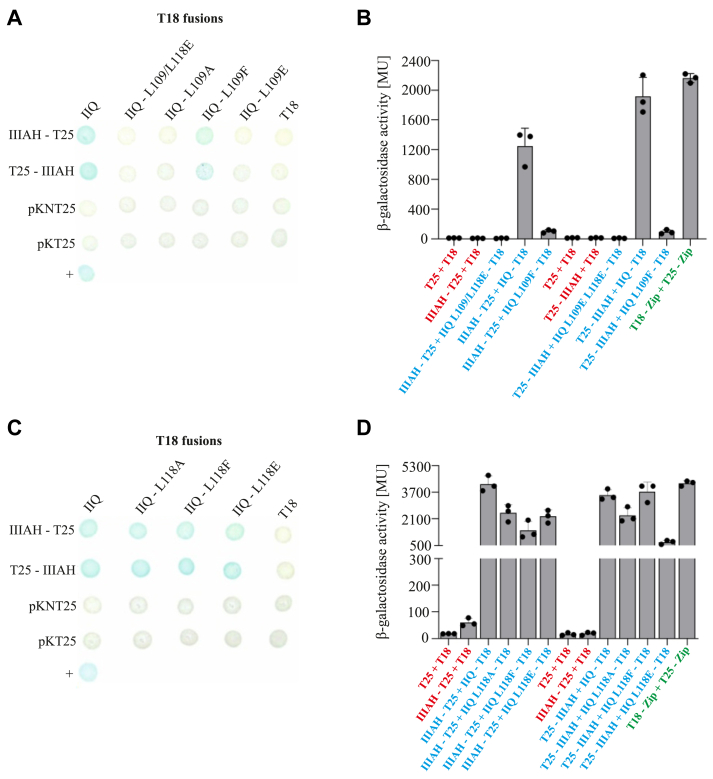
**Interactions of the extracytoplasmic component of SpoIIIAH with the extracytoplasmic component of the SpoIIQ variants**. *A* and *C*, BACTH showed different interaction properties of SpoIIQ variants. *E*. *coli* strain BTH101 (*Δcya*) was cotransformed with plasmids encoding the indicated fusions to adenylate cyclase fragments T18 and T25. Colonies were spotted on selective plates containing IPTG and X-Gal. The *blue color* indicates a positive interaction between the specified pair of fusion proteins. The positive control, T18-Zip + T25-Zip, is marked +. Fusions of the extracytoplasmic part of the SpoIIQ variants with fragment T25 are shown in supporting information (Fig. S1). *B* and *D*, BACTH β-galactosidase activity assays. Normalized β-galactosidase activity expressed in Miller units (MU) is shown. The mean values from each experiment were normalized to the negative-control (in *red*) values (BTH101 cells coexpressing only T18 and T25 subunits of adenylate cyclase). The values of specific negative controls (only one subunit of adenylate cyclase fused with the respective target; the second subunit remained free) are also shown. Each experiment was performed at least three times independently. The bars represent averages, and the dots represent individual experiments. Error bars represent ±SD. *Red* and *green* text in the x-axis description indicates negative (T25 + T18, IIIAH-T25 + T18) and positive controls (T18-Zip + T25-Zip), respectively. Note that some negative interactions from (*A*) and (*C*) are not included in this assay. BACTH β-galactosidase activity assays of fusions of the extracytoplasmic part of SpoIIQ variants with fragment T25 are shown in supporting information (Fig. S1).

To assay protein-protein interactions involving the SpoIIQ mutant variants and SpoIIIAH we employed a bacterial two-hybrid (BACTH) system (24). We tested the interactions using constructs in which fragments of adenylate cyclase were fused to either the N- or the C-terminus of each protein. Interaction of the fused fragments leads to reconstitution of adenylate cyclase activity, production of cyclic AMP, and expression of the reporter gene coding for β-galactosidase. We confirmed that for the wild type proteins, the extracytoplasmic domain of SpoIIQ interacts with the extracytoplasmic domain of SpoIIIAH in this assay as evidenced by the blue color of the spots in Figure 2. This is in agreement with the previously described interaction of these proteins (13). We then tested if mutations in SpoIIQ that contribute to the SpoIIIAH binding surface cause changes in the interacting properties of SpoIIQ. We found that mutations L109 A, L109 E and the double mutation L109E-L118 E (L109/118E) led to the loss of interaction with SpoIIIAH (Fig. 2, *A* and *C*, Fig. S1). Mutations L109 F (Fig. 2, *A* and *C*, Fig. S1) and all three mutations of L118 did not appear to change the ability of SpoIIQ to interact with SpoIIIAH (Fig. 2, *B* and *D*, Fig. S1). The more sensitive measurement of β-galactosidase activity in cell lysates of these strains, however suggested that the interactions of SpoIIQL109F (Fig. 2, *A* and *C*, Fig. S1) and all three L118 mutants with SpoIIIAH are weaker than the interaction of the wild type protein (Fig. 2, *B* and *D*, Fig. S1). Taken together, these results indicate that the L109 mutations have more drastic effects relative to the L118 mutations. Consequently, we focused on characterizing these mutations in more detail.

### Extracytoplasmic domains of SpoIIQL109/118E and SpoIIIAH do not interact in an *in vitro* pull-down assay

**Figure 3:**
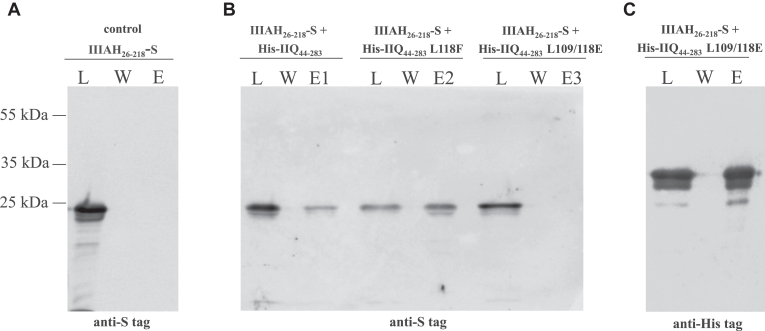
**Interaction of the extracytoplasmic domains of SpoIIIAH and SpoIIQ variants tested by pull-down assay**. *A*, Control for non-specific binding of SpoIIIAH_26-218_-S to the Ni^2+^-chelation column (IIIAH_26-218_-S). Soluble protein extract from cells expressing the extracytoplasmic domain of SpoIIIAH-S alone loaded on to the column (L), final wash fraction (W) and eluate (E). *B*, His-SpoIIQ_44-283_ (His-IIQ_44-283_) and His-SpoIIQL118F_44-283_ (His-IIQL118F_44-283_) pull down SpoIIIAH_26-218_-S from *E.coli* cell lysates on a Ni^2+^-chelation column (*panelB*, lanes E1, E2). His-SpoIIQL109/118E_44-283_ (His-IIQL109/118E_44-283_) does not pull down SpoIIIAH_26-218_-S from *E.coli* cell lysates on the Ni^2+^-chelation column (*panel B*, lane E3). Western blotting using anti-S tag monoclonal antibody (*panelB*) and anti-His tag monoclonal antibody (*panelC*) was used to detect fused proteins in soluble load fractions (L), final wash fractions (W) and eluates (E). A selected part of the protein ladder is shown to the *left* of the Western blots. The size of detected proteins is SpoIIIAH_26-218_-S – 23.5 kDa and His-SpoIIQ_44-283_ – 27.5 kDa, respectively.

To corroborate the BACTH observations, we performed *in vitro* pull-down experiments to monitor the association of SpoIIQ and its mutant variants with SpoIIIAH. For this purpose, wild type and site directed mutant variants of the extracytoplasmic domain of SpoIIQ were produced as His-tag fusions (His-SpoIIQ_44-283_). Meanwhile, the extracytoplasmic domain of SpoIIIAH was produced as an S-tag fusion (SpoIIIAH_26-218_-S). The expression and solubility of these fusion proteins in *E*. *coli* extracts was confirmed by SDS-PAGE. For interaction studies mixtures of extracts containing the wild type or mutant His-SpoIIQ_44-283_ proteins and extracts containing SpoIIIAH_26-218_-S were loaded onto a Ni^2+^-chelation column. In this assay, His-SpoIIQ_44-283_ would be affinity captured on a Ni^2+^-chelation column and interacting SpoIIIAH_26-218_-S would then be co-captured, co-eluted and subsequently detected using its fused S-tag. We confirmed direct interaction between the extracytoplasmic domain of SpoIIIAH and both wild-type SpoIIQ and a mutant in which the leucine in position 118 was substituted by phenylalanine (L118F). In contrast, we affirmed that the SpoIIQ double mutant (L109/118E) does not interact with SpoIIIAH (Fig. 3, *B* and *C*). To test for nonspecific binding of SpoIIIAH_26-218_-S to the column, extracts expressing SpoIIIAH_26-218_-S alone were loaded onto a Ni^2+^ column as a control. We did not detect S-tagged SpoIIIAH_26-218_ in elution fractions when it was produced alone (Fig. 3*A*).

### SpoIIQ mutant strains differ in their sporulation efficiency and SpoIIQ localization patterns

**Figure 4:**
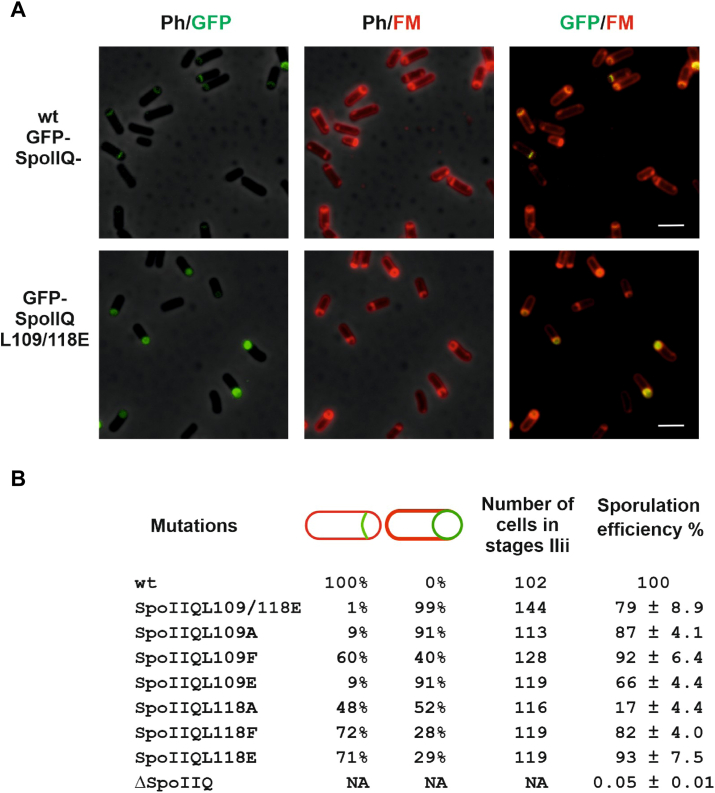
**Characterization of SpoIIQ mutant strains**. *A*, localization of SpoIIQL109/118E. Sporulating cultures of strains IB1856 (mGFP-SpoIIQ) and IB1857 (mGFP-SpoIIQL109/118E) were observed by fluorescence microscopy. Cells were induced to sporulate by exhaustion in DSM medium and images were taken 2 h after the onset of sporulation. Images in the first column: overlay of phase contrast with mGFP fluorescence; images in the second column: overlay of phase contrast with membranes visualized using FM 4 to 64; images in the third column: overlay of GFP and membranes. The scale bars represent 3 μm. *B*, localization of SpoIIQ mutants in stage IIii of sporulation (curved sporulation septa). The first column shows mutation in SpoIIQ; wt represents mGFP-SpoIIQ. The next panel shows the fraction of cells in stage IIii in which mutant mGFP-SpoIIQ was either at the polar septum or diffused predominantly in the forespore membrane. The fourth column shows the total number of evaluated cells in stage IIii. The far-right column gives the sporulation efficiency of mutant strains determined through a heat resistance assay and reported as relative to wild type IB1856 (mGFP-SpoIIQ).

Previous localization experiments revealed that early in sporulation GFP-SpoIIQ localizes to the asymmetric septum (25). Later SpoIIQ tracks the engulfing mother cell membrane, assembling in helical arcs and foci surrounding the forespore. After completion of membrane fusion, GFP-SpoIIQ is degraded and fluorescence is observed in the forespore cytoplasm (25). To follow localization of SpoIIQ mutant forms, we prepared strains in which SpoIIQ variants, encoded at an ectopic *amy* locus, were fused to sequences encoding mGFP (IB1856–1863). The fusions were expressed under the control of the native promoter as the sole source of SpoIIQ. The sporulation efficiency of these strains was determined revealing that with the exception of the strain carrying the L118 A mutation (17% sporulation efficiency compared to wild type) all strains exhibit only slightly lower levels of heat-resistant spore formation than the wild-type strain (Fig. 4*B*, Table S1).

**Figure 5:**
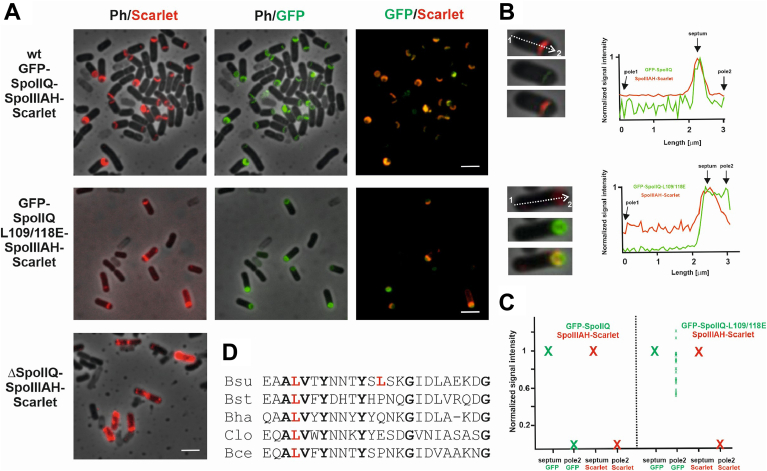
**SpoIIIAH localization in SpoIIQL109/118E strai**n. *A*, SpoIIIAH-mScarlet localizes at the polar septum in the mGFP-spoIIQL109/118E double mutant strain in the same way as it does in the wild type strain. Images in the first column are an overlay of phase contrast (Ph) and Scarlet fluorescence; images in the second column are an overlay of phase contrast and GFP fluorescence; and images in the third column are an overlay of Scarlet and GFP. The scale bars represent 3 μm. *B*, signal intensity of GFP-SpoIIQ and SpoIIIAH-Scarlet. Distribution of fluorescence signal intensity along the long axis of the wt and *spoIIQL109/118E* double mutant cells. The three images for the wt and double mutant are an overlay of phase contrast with Scarlet fluorescence; an overlay of phase contrast with GFP fluorescence; and an overlay of phase contrast, Scarlet and GFP. *C*, the signal intensities of GFP-SpoIIQ and SpoIIIAH-Scarlet. We determined the signal intensities of these proteins at the asymmetric septum and at the forespore cell pole (pole 2) for 20 different cells. For each cell, we set the signal intensity of SpoIIIAH-Scarlet at the septum to 1 and the signal intensity of GFP-SpoIIQ at pole 2 to 0. The red “X“ represents SpoIIIAH-Scarlet and the green “X“ represents GFP-SpoIIQ. The lower case green “x” represents the ratio of the signal intensity of GFP-SpoIIQL109/L118 E at cell pole 2 to its signal intensity at the septum for 20 individual cells. *D*, sequence alignment of L109-L118 regions of SpoIIQ homologues from *B*. *subtilis* (Bsu), *B*. *stearothermophilus* (Bst), *B*. *halodurans* (Bha), *Clostridioides difficile* (Clo) and *B*. *cereus* (Bce). L in red represents leucines (Leu 109 and Leu 118 in *B*. *subtilis*) that were mutated and letters in bold represent invariant residues in all five compared species.

Localization of the GFP-SpoIIQ mutant forms was examined 2 h after the onset of sporulation. GFP-SpoIIQ in stage IIii (curved sporulation septa) assembles in arcs matching the curvature of the septum as was shown previously (25). Mutant SpoIIQ variants show differing patterns of localization (Fig. 4*A*); SpoIIQL109/118E, SpoIIQL109 A and SpoIIQL109E signals were predominantly diffuse throughout the forespore membrane (Fig. 4*B*). All other SpoIIQ mutants were partially correctly localized at this stage with 48% to 72% of cells exhibiting a curved pattern of localization that matches the curvature of the septum (Fig. 4*B*). Immunoblot analysis of GFP-SpoIIQ showed no significant differences in GFP-SpoIIQ protein levels between the *B*. *subtilis* wild-type and GFP-SpoIIQL109/118E mutant strains (Fig. S2*B* Lanes 2 and 3). However, the double SpoIIQ mutant (GFP-SpoIIQL109/118E) undergoes increased degradation compared to the wild-type protein (Fig. S2*B* Lanes 2 and 3), and thus, it is possible that this influenced, but only partially, the mislocalization of the signal as presented in Figures 4, *A* and *B* and 5*C*. We also checked, by Western blot analysis, the GFP-SpoIIQL118 E single mutant strain. Here we observed similar amounts of degradation products to those observed for the double mutant (Fig. S2*B* Lanes 3 and 4). The single mutant protein localizes to the sporulation septum in 71% of the cells in stage IIii in striking contrast to the double mutant protein which localizes to the septum in only 1% of the cells at this stage (Fig. 4*B*). Thus, it is unlikely that the large difference in the GFP localization pattern in the double mutant strain can be explained by GFP-SpoIIQL109/118E degradation.

### SpoIIIAH localization is not affected in the SpoIIQL109/118E mutant strain

Previous localization experiments showed that in cells that have initiated engulfment, SpoIIIAH localizes preferentially to the polar septum as it develops a slight curvature (13, 26). Later SpoIIIAH tracks the engulfing mother cell membrane as it moves around the forespore. In engulfed sporangia, SpoIIIAH localizes in discrete foci around the forespore (13). Immunofluorescence microscopy showed that SpoIIQ colocalizes with SpoIIIAH (13). Thus during engulfment, SpoIIQ-myc and SpoIIIAH-Flag, form overlapping fluorescent foci along the engulfing membrane. Later, while many SpoIIQ-myc foci continued to colocalize with SpoIIIAH-Flag foci, other foci were not colocalized, probably due to the instability of SpoIIQ-myc (13). SpoIIIAH is randomly distributed throughout the mother cell membrane in the absence of SpoIIQ, indicating that SpoIIIAH localization depends on SpoIIQ (13).

It was therefore of interest to test the effects on H-Q colocalization of our loss-of-interaction SpoIIQ double mutant L109/118E. As this double mutation has the most potent effects on the localization characteristics of SpoIIQ, we chose to monitor SpoIIIAH localization in this mutant background. To provide a basis for colocalization studies in the *spoIIQ* mutant strains, we initially prepared strain KM1600 in which the sequence coding for SpoIIIAH at its native locus was fused to sequence encoding mScarlet. The fusion is expressed under the control of the native promoter, *p*_*spoIIIAA*_. This strain produced wild-type levels of spores (Table S2), indicating that SpoIIIAH-mScarlet was functional. To follow the localization of SpoIIQL109/118E and SpoIIIAH simultaneously, we prepared strain KM1602 as a derivative of KM1600 in which mGFP-SpoIIQL109/118E is produced. We observed that even though mGFP-SpoIIQL109/118E is distributed throughout the forespore in cells in stage IIii, SpoIIIAH-mScarlet localizes at the polar septum and forms an arc matching the curved sporulation septum seen in wild type cells (Fig. 5*A*). Analysis of the fluorescence signal distribution along the long axis of the cell revealed that in strain KM1601 (GFP-SpoIIQ + SpoIIIAH-mScarlet) both the GFP and Scarlet signals exhibited a single, distinctive peak at the septum (Fig. 5, *B* and *C*), confirming colocalization of SpoIIQ and SpoIIIAH at this site. In the mutant strain KM1602, (GFP-SpoIIQL109/118E + SpoIIIAH-mScarlet) GFP signal displayed one peak at the septum and an additional peak at the adjacent cell pole 2; the Scarlet signal intensity in the mutant peaked at the septum (Fig. 5*B*). GFP-SpoIIQ/L109/118E localization differs from the localization of wild type GFP-SpoIIQ at the cell poles. In the mutant cells (n = 20) GFP signal intensities at the poles were at levels 0.5 to 1.2 in comparison to wild type cells (n = 20) where they were at the background level (Fig. 5*C*). In a *ΔspoIIQ* background SpoIIIAH-mScarlet is distributed in the mother cell membrane (Fig. 5*A*) which is in good agreement with previous results (13).

### Measurement of σ^G^ activity showed lower activity in spoIIQ mutant strains

**Figure 6:**
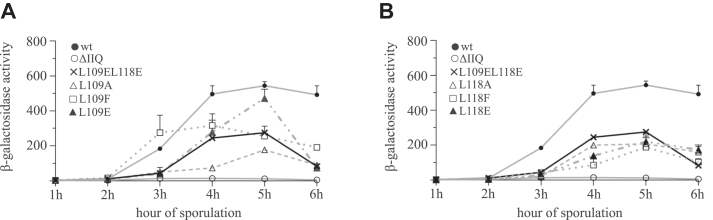
**Monitoring σ^G^-dependent β-galactosidase activity from p_sspE_-lacZ fusion**. Cells were induced to sporulate by nutrition exhaustion in DSM medium. *A*, β-galactosidase activity from p_sspE_-lacZ fusion in strains with specified mutations of L109 in SpoIIQ. *B*, β-galactosidase σ^G^-specific activity from p_sspE_-lacZ fusion in strains with specified mutations of L118 in SpoIIQ. wt represents the KM1557 strain (Table S3). The symbols represent averages from three independent experiments; error bars represent ±SD.

Activation of the late-acting forespore-specific sigma factor, σ^G^, requires the production of the eight mother cell proteins SpoIIIAA-AH and the forespore protein SpoIIQ (17). SpoIIIAA-AH are products of the *spoIIIA* operon that is transcribed under the control of σ^E^ (27) and SpoIIQ is produced by σ^F^ directed transcription of the *spoIIQ* gene (9, 11). It has been proposed that a complex of the SpoIIIAA-AH proteins and SpoIIQ proteins forms a channel connecting the mother cell and the forespore (16, 17, 18, 21, 28). This channel is proposed to serve as a feeding tube through which the mother cell nurtures the developing spore by providing small-molecule metabolites for macromolecular synthesis in the forespore (17, 29). Formation of this complex/channel is required to maintain transcriptional potential in the forespore, including σ^G^ activity, however how this is achieved is still not clear (10, 16, 17, 29). It was demonstrated previously that *spoIIQ* null mutants almost completely lack σ^G^ activity (9). Therefore, we wanted to find out if mutations in SpoIIQ that disrupt the H-Q interface affect σ^G^ activity. To measure σ^G^ activity, we introduced a p_sspE_-lacZ fusion that is under the transcriptional control of σ^G^ (11) into strains expressing mutant SpoIIQ (IB1856–1863) resulting in strains KM1557 to 1567 (Table S3). The σ^G^ activity inferred from the β-galactosidase activity of the p_sspE_-lacZ fusion revealed that in all strains except for the L109 F mutant, the peak in activity was reached at the fifth hour after the initiation of sporulation, which is in good agreement with previous measurements (11). Strikingly, σ^G^ activity in all of the mutant strains was lower than in the strain producing wild type SpoIIQ (Fig. 6). Despite their lower σ^G^ activity, these strains sporulate efficiently (Fig. 4*B*, Table S1).

## Discussion

**Figure 7:**
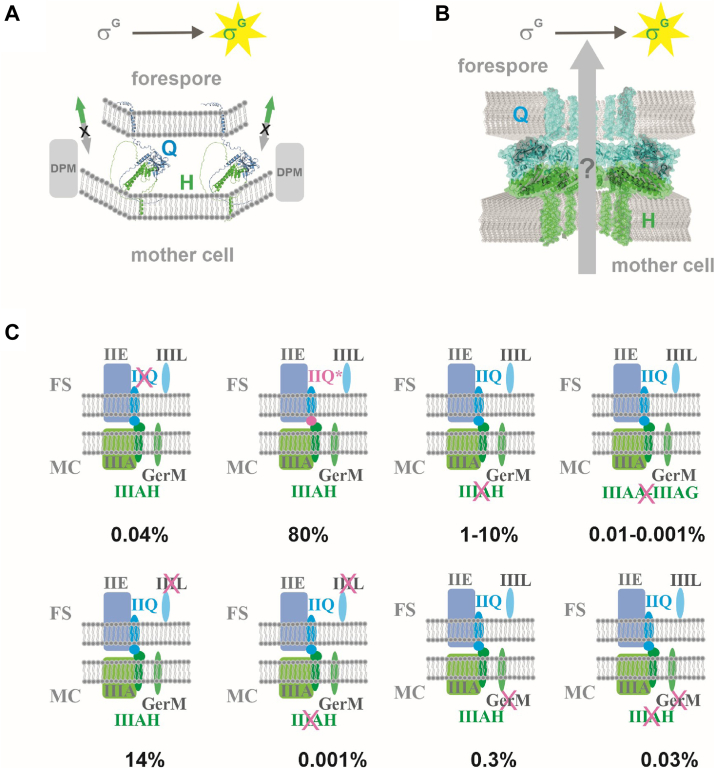
**Model for the role of SpoIIIAH-SpoIIQ interaction in the activation ofσ^G^ in the forespore**. *A*, Zipper formation between the forespore protein SpoIIQ and mother cell SpoIIIAA-AH is required for σ^G^ activity. This multiprotein complex functions as a ratchet that prevents backward movement of the engulfing membrane (marked as X) and makes forward membrane movement irreversible (*green arrows*). SpoIID-SpoIIP-SpoIIM (DPM) complex advances the leading edge of the engulfing membrane with SpoIIIAA-AH and SpoIIQ protein complex (for simplicity marked as H and Q) zipping up behind. *B*, model of channel formation. View of the modeled H-Q assembly, showing additional transmembrane helices in the intercellular space and pores in the respective membranes that allow the passage of yet-unidentified factors required for σ^G^ activity (adapted from (19). "H" represents SpoIIIAH and "Q" SpoIIQ proteins, respectively. *C*, percentage sporulation efficiencies of mutant strains relative to wild type. IIQ represents SpoIIQ, IIQ∗ - SpoIIQ mutant versions, IIIL - SpoIIIL, IIE – SpoIIE and IIIA – SpoIIIAA-AG. Crosses indicate deletion of genes coding for the corresponding proteins. The cross for IIIA-G represents the deletion of all single genes coding for SpoIIIAA, SpoIIIAB, SpoIIIAE, SpoIIIAF and SpoIIIAG. The mother cell specific proteins are in different *green shades* and forespore proteins are in *blue shades*. The mother cell is marked MC and forespore FS.

Endospore formation in *B*. *subtilis* is associated with a complex process called engulfment. It begins with the localization of participating proteins to the asymmetric septum and their assembly into engulfment complexes capable of remodeling peptidoglycan and mediating migration of the enveloping cell membrane around the target cell membrane. It was proposed that the forespore protein SpoIIQ and the mother cell protein SpoIIIAH bridge these two adjacent cells, interacting through their extracytoplasmic domains across the intermembrane space (13). In the final step, the leading edges of the enveloping membranes meet and undergo fission, releasing the forespore into the mother cell as a cell-within-a-cell. The function of the *spoIIIA* and *spoIIQ* genes in engulfment has long been of great interest (30). Different, but not mutually exclusive, models of how this H-Q complex works invoke zipper-like and mechanical ratchet systems (14), as well as a protein channel (17, 18, 19) (Fig. 7, *A* and *B*).

In this work, we investigated the importance of H-Q interactions by analysis of mutations in SpoIIQ located in its SpoIIIAH binding surface. Two leucine residues on SpoIIQ, L109 and L118, which are buried in the SpoIIIAH interface in the crystal structure were individually substituted by alanine, phenylalanine and glutamate. In addition, we prepared a double mutant in which both leucines were replaced by glutamates. Using a BACTH system, we showed that the SpoIIQ mutants with L109A, L109E and the double L109/118E substitution, do not interact with SpoIIIAH. An *in vitro* pull-down assay further confirmed that double mutant L109/118E does not interact with SpoIIIAH. The single L109 mutations had more pronounced effects on the H-Q interaction surface than those at L118. This was not initially predicted from the crystal structures but might have been anticipated from sequence comparisons (Fig. 5*D*) which show invariance of Leu109 but divergence of Leu118 among SpoIIQ orthologues from sporulating bacteria. Retrospective consideration of the structure shows that the distal part of the Leu109 side chain forms more, and closer, contacts with the main chain of SpoIIIAH than Leu118 whose intermolecular interactions feature side chains. The latter can presumably more easily alter their conformation to accommodate the substitutions introduced here.

To measure the effect of *spoIIQ* mutations *in vivo*, we analyzed the localization of mutant SpoIIQ proteins in *B*. *subtilis*. Septal localization of SpoIIQ requires the σ^E^-controlled protein SpoIIIAH, degradation of septal peptidoglycan, and the mother cell protein GerM (31). In cells harboring the SpoIIQ variants (SpoIIQL109/118E, SpoIIQL109 A, and SpoIIQL109 E), that did not interact with SpoIIIAH in our BACTH assay (Fig. 2, *A* and *B*), SpoIIQ in stage IIii was distributed throughout the forespore membrane (Fig. 4*A*). Additional analysis of the fluorescence signal distribution along the long axis of the cell revealed two distinct peaks for GFP-SpoIIQL109/118E: one at the septum, as was observed for wild type GFP-SpoIIQ, the other at the adjacent cell pole which is never observed in wild type GFP-SpoIIQ (Fig. 5, *B* and *C*). The observed localization of GFP-SpoIIQL109/118E at the cell pole indicates that A-Q interaction is largely disrupted in live cells. However, we also observed that in the majority of cells, GFP-SpoIIQL109/118E signals are stronger at the septum than at the poles (Fig. 5*C*). This may indicate that the DPM complex, GerM and/or another, not yet identified, partner of SpoIIQ are sufficient to ensure, at least partially, correct localization of mutant SpoIIQ even in the absence of direct interaction with SpoIIIAH. In addition, in these cells SpoIIIAH localizes to the curved polar septum as it does in wild-type cells (Fig. 5, *A* and *B*, *C*). Together, these observations indicate that SpoIIQ is important for SpoIIIAH localization at the septum by a mechanism that does not depend on direct interaction of the two proteins. The finding that cells with mutated SpoIIQ despite having lower σ^G^-specific activity (Fig. 6), sporulate efficiently (Fig. 4*B*, Table S1), suggests that SpoIIQ’s essential function is not dependent on its interaction with SpoIIIAH.

One of the proposed models for how H-Q functions invokes zipper-like interactions between SpoIIIAH and SpoIIQ that form a ratchet which prevents backward movement of the engulfing membrane (Fig. 7*A*) (14). The contributions of SpoIIQ and SpoIIIAH to engulfment are essentially redundant in wild-type cells where their function is masked by other engulfment modules such as the SpoIID-SpoIIP-SpoIIM (DPM) complex (Fig. 7*A*). The DPM complex mediates septal thinning and is rate-limiting for membrane migration (32). Engulfment is thus successfully completed in our mutant cells, even though SpoIIQ interactions with SpoIIIAH are compromised.

The second proposed model of an intercellular channel during engulfment was based on experiments suggesting that the forespore and mother cell cytoplasms are connected (18) and able to exchange small molecules (16). This idea was augmented with the discovery that the *spoIIIA* operon encodes components that resemble type III secretion system proteins (18). It is proposed that an intercellular bridge is formed by interactions between SpoIIIAH and SpoIIQ similar to those observed in crystal structures of their complexes (22, 23). These complexes comprised the extracytoplasmic domains alone of the respective proteins which form heterodimers. Although plausible models of multimeric H-Q circular assemblies were derived from these heterodimers, these assemblies remain uncorroborated.

The results presented here challenge aspects of this channel model. The L109/118E double substitution in the proposed SpoIIIAH-interacting surface of SpoIIQ disrupts H-Q complex formation. Surprisingly, sporulation in these cells is essentially unaffected (80% efficiency relative to the wild-type strain). This implies either (1) that the integrity of the channel is not essential for sporulation or (2) that interactions other that those between SpoIIQ and SpoIIIAH can hold the channel together. In the former case, it is conceivable that solutes can pass out of the mother cell through a SpoIIIAA-AH complex into the intercellular space and subsequently diffuse through a SpoIIQ pore without the two components being physically attached.

More broadly, the H-Q channel was not observed by cryo-electron tomography of *B*. *subtilis* sporangia (33). Moreover its role in the activation of the forespore specific sigma factor σ^G^ is unclear. Similarly, the nature and range of molecules transported through the channel is yet to be established. One study showed that the SpoIIIAA-AH:SpoIIQ complex allows the fluorescent dye calcein to move in both directions between the mother cell and the forespore and it was recently shown that nucleoside di- or triphosphates can be transported from the mother cell to the forespore (29, 34) as part of metabolic nurturing. As the SpoIIIAA-AH:SpoIIQ complex dissociates shortly after the end of engulfment (35), there are some doubts about its capacity to sustain feeding of the forespore by the mother cell (30).

SpoIIQ is required for efficient sporulation and necessary for activation of the late forespore sigma factor, σ^G^ (8, 9). Since cells producing SpoIIQ mutants deficient in SpoIIIAH interactions have negligible sporulation defects, interactions of SpoIIQ with other partner proteins are presumably essential for spore formation (Fig. 7*C*). SpoIIQ was shown to interact directly with SpoIIE (36). However, the significance of this interaction was clarified only recently; it was shown that SpoIIQ-mediated localization of SpoIIE to the septal membranes contributes to the stabilization of the septum and plays a significant role in compartmentalization at the onset of engulfment (37).

Another potential SpoIIQ partner was discovered in a high-throughput screen for additional sporulation genes (38). GerM is a mother cell protein expressed under the control of σ^E^, which is required for the localization of SpoIIQ in the septal membrane (31). A *gerM* mutant has a synergistic sporulation defect with *spoIIIAH*; the *ΔspoIIIAHΔgerM* double mutant has a sporulation efficiency of 0.03%, like that of a *ΔspoIIQ* strain (Fig. 7*C*). GerM localization to the septum requires peptidoglycan hydrolysis and SpoIIQ. In addition, GerM in the absence of all other σ^E^ dependent genes, is sufficient to localize SpoIIQ (provided the cell wall has been sufficiently thinned). These data indicate that GerM is a component of the H-Q complex, which may constitute the basal platform for localizing other sporulation proteins on both sides of the septal membrane (31). A second sporulation protein discovered in the high-throughput screen and implicated as part of the H-Q complex is the forespore protein SpoIIIL (38). SpoIIIL is a cytoplasmic protein expressed under the control of σ^F^. Cells lacking SpoIIIL had a sporulation efficiency of 14% compared to the wild type (Fig. 7*C*). Subpopulations of these cells had smaller forespores and often reduced σ^G^ activity. In addition, a *ΔspoIIILΔspoIIIAH* double mutant displays a synergistic phenotype with a sporulation efficiency of 0.001% compared to the wild type (Fig. 7*C*) (38). Recent cytological, genetic and biochemical analysis however revealed that SpoIIIL is not a part of the SpoIIIAA-AH:SpoIIQ complex (39) as was assumed previously (38). Instead, SpoIIIL is thought to contribute to the cell-cell signalling pathway that activates σ^K^ in the mother cell. SpoIIIL may function as an activator of SpoIVB protease, which is essential for proteolytic processing, and activation of σ^K^ in the mother cell (39). Another genetic screen identified several previously uncharacterized genes involved in envelope remodelling during the engulfment stage of sporulating *B*. *subtilis* cells (40). Among these, the MurA paralog MurAB, implicated in peptidoglycan precursor synthesis, was discovered. MurAB contributes to efficient engulfment in cells lacking SpoIIIAH, indicating that it is additionally required when the SpoIIIAH-SpoIIQ ratchet is abolished (40).

In summary, SpoIIQ and SpoIIIAH are components of a multiprotein complex that includes SpoIIE, SpoIIIL, SpoIIIAA-AG and GerM. In the absence of direct H-Q interaction, other SpoIIQ functions and/or interactions allow activation of σ^G^ and efficient sporulation (Fig. 7*C*). According to the revised model in Figure 7, multiprotein complexes from the mother cell and the forespore sides of the sporulation septum maintain the zipper-like interaction between both complexes even in the absence of H-Q interaction. However, the H-Q channel is not a feasible mechanism for how SpoIIIAH and SpoIIQ protein’s function. Nevertheless, we cannot exclude the possibility that SpoIIQ and SpoIIIAH are involved in multiple close-packing interactions with other proteins in the assembly of a putative channel through other SpoIIIA proteins such SpoIIIAG and SpoIIIAF which has been observed as oligomeric rings (20, 21). Knowledge of the structure and assembly of all the proteins involved is essential for understanding their integrated function in reshaping the cell membrane and the cell wall and allowing the fascinating engulfment process during bacterial endospore formation.

## Experimental procedures

### Bacterial strains and plasmids

*E*. *coli* strains were grown in LB broth (41). *B*. *subtilis* cells were grown in DSM (42). When required, media were supplemented with 5 μg ml^-1^ chloramphenicol, 100 μg ml^-1^ spectinomycin, 1 μg ml^-1^ erythromycin and 25 μg ml^-1^ lincomycin. In general, molecular biology experiments and measurement of sporulation efficiency in *B*. *subtilis* were carried out as described previously (42).

The bacterial strains used in this study are listed in Table S3; plasmids used in this study are listed in Table S4; sequences of the oligonucleotides used in this work are given in Table S5.

To construct an mGFP-spoIIQ fusion plasmid (pSGmgfp-IIQ) PCR fragments containing *spoIIQ* promoter, *mGFP* and *spoIIQ* coding sequences were inserted into SpeI, HindIII digested pSG1154 (43) by isothermal assembly. The construct was used to transform *B*. *subtilis* MO1099 (44) and the resulting transformants were confirmed to be the product of double crossover recombination.

To prepare constructs with selected mutations in *spoIIQ*, a 2 kb p_spoIIQ_-mGFP-spoIIQ PCR fragment was amplified from chromosomal DNA from strain IB1848 and cloned into pCR-Blunt using the Zero Blunt PCR cloning kit (Invitrogen). The recombinant plasmid pCR18 was used as the template for mutagenesis of the SpoIIQ core residues Leu109 and Leu118 to Ala, Phe, and Glu using the Q5 site directed mutagenesis kit (NEB). The mutated plasmids were sequenced to confirm the presence of the mutation, then digested with SpeI and HindIII and the resulting 2 kb fragments were inserted into similarly digested pSG1154 (43). The recombinant plasmids were used to transform *B*. *subtilis* MO1099 (44) and the transformants arising were confirmed to have inserts resulting from double-crossover recombination. To construct pSGcspoIIIAH-mscarlet 444 nucleotides from the 3′ end of the *spoIIIAH* coding sequence (omitting the stop codon) were PCR amplified using the primers cIIIAHKpnS and cIIIAHKpnE and ligated into the vector pSGrefZ-mscarlet (40), so as to create a fusion of the SpoIIIAH C-terminal domain to mScarlet. The resulting plasmid was then used to transform *B*. *subtilis* PY79 (45).

To analyze the interaction between the extracytoplasmic domains of SpoIIQ (44–283 aa) and SpoIIIAH (26–218 aa) by pull-down methods, pETIIQ or its mutant variants (pETIIQL109/118E and pETIIQL118 F) and pETIIIAH were constructed. PCR fragments containing the extracytoplasmic part of the *spoIIQ* (or *spoIIQ*L109/118E and *spoIIQ*L118 F, respectively) were prepared using the primers IIQextraBamF5 and IIQextraEcoRstop. To yield pETIIQ (or mutant variants), these PCR fragments were digested with BamHI and XhoI and cloned into similarly cut pETDuet-1 vector. To construct pETIIIAH, a PCR fragment containing the extracytoplasmic domain of *spoIIIAH* was amplified using the IIIAHextra_Stag_NdeF and IIIAHextra_Stag_XhoR primers and, after digestion with NdeI and XhoI, was cloned into a similarly digested pETDuet-1 vector.

### Bacterial two-hybrid system and quantitative β-galactosidase assay

Sequences encoding *spoIIQ*, *spoIIIAH* and truncated forms specifying their extracytoplasmic domains were PCR amplified using combinations of primers listed in Table S5. PCR fragments were digested with BamHI and EcoRI and cloned into the vectors of a BACTH bacterial two-hybrid system (24) to generate plasmids encoding fusions to the T25 and T18 fragments of adenylate cyclase. To test for protein-protein interactions, pairs of plasmids were co-transformed into *E*. *coli* BTH101. Co-transformation mixtures were spotted onto LB plates supplemented with 40 μg ml^−1^ X-Gal (5-bromo-4-chloro-3-indolyl-β-d-galactopyranoside), 0.5 mM isopropyl β-D-thiogalactoside (IPTG), 100 μg ml^−1^ ampicillin and 30 μg ml^−1^ kanamycin, and grown for 24 to 48 h at 30 °C. β-galactosidase activity was measured as described by Miller (46) with the inclusion of an extra wash step.

### Protein isolation and purification

The expression plasmids pETIIQ (or pETIIQL109/118E and pETIIQL118 F, respectively) and pETIIIAH prepared as described above were transformed into *E*. *coli* BL21 (DE3) strain. Strains harboring these expression plasmids were grown in LB medium at 37 °C to an OD_600_ of 0.6. Expression of recombinant proteins was induced by addition of 1 mM IPTG. After 4 h of further growth at 37 °C the cells were harvested by centrifugation at 3000 rpm for 20 min. Cell pellets from 12.5 ml of cultures of His-SpoIIQ_44-283_ (or its mutant variants), and cell pellets from 25 ml of SpoIIIAH_26-218_-S, respectively, were resuspended in lysis buffer (50 mM Tris-HCl pH 8.0, 150 mM NaCl, and 1 mM 4-(2-aminoethyl) benzenesulfonyl fluoride [AEBSF]). For interaction studies, cells producing the wild type or mutant His-SpoIIQ_44-283_ proteins were mixed with cells producing SpoIIIAH_26-218_-S and lysis was achieved by sonication. The lysates were centrifuged at 30,000 rpm for 30 min to remove cell debris and loaded onto 1 ml Ni Sepharose HP columns (GE HealthCare). After 10 washing steps each time with 1 ml of buffer containing 40 mM imidazole proteins were eluted with 1 ml of 1 M imidazole. Co-eluted proteins were identified by Western blot analysis using monoclonal antibodies against the His-tag (cat. number 70796–3, Lot: 3,172,550, Novagen) or the S-tag (cat. number 71549–3, Lot: 3,118,452, Novagen).

### Immunoblot analysis

Samples of solubilized *B*. *subtilis* membrane proteins were prepared as previously described (36), with minor modifications. Briefly, 100 ml cultures were harvested 2.5 h after the onset of sporulation and washed twice with 1 × SMM buffer (0.5 M sucrose, 20 mM MgCl_2_, 20 mM maleic acid, pH 6.5) at room temperature. Cells were treated with lysozyme (0.5 mg ml^−1^), and the protoplasts were collected by centrifugation and frozen in liquid nitrogen. Thawed protoplasts were disrupted by osmotic lysis with 3 ml hypotonic buffer (buffer H) (20 mM HEPES pH 8.0, 200 mM NaCl, 1 mM dithiothreitol, 1 mM MgCl_2_, 1 mM CaCl_2_ supplemented with protease inhibitors (cOmplete Protease Inhibitor Cocktail tablets, Sigma, 1 mM AEBSF, Sigma) and lysates were treated with DNAse I (10 μg ml^−1^) (Sigma) and RNAseA (20 μg ml^−1^) (Sigma) for 1 h on ice. The membrane fraction was separated by centrifugation at 30,000 rpm for 1 h at 4 °C. Subsequently the membrane pellet was resuspended in 200 μl of buffer G (buffer H with 10% glycerol) and crude membranes were aliquoted and frozen. 100 μl crude membranes were diluted 3-fold with buffer S (buffer H with 20% glycerol and 100 μg ml^−1^ lysozyme), and membrane proteins were solubilized by the addition of the nonionic detergent digitonin (Sigma) to a final concentration of 0.5%. The mixture was rotated at 4 °C for 1 h. Soluble and insoluble fractions were separated by centrifugation at 30,000 rpm for 1 h at 4 °C. The soluble fractions from the digitonin treated membrane preparations were used for immunoblot analysis. Proteins fused with GFP were identified by Western blot analysis using monoclonal antibody against GFP (mouse monoclonal [9F9.F9] to GFP, ab1218 Abcam).

### Monitoring σ^G^-directed β-galactosidase activity

A single colony from each *B*. *subtilis* strain was resuspended in 100 μl of LB medium, spread onto a LB plate and grown overnight at 30 °C. This overnight culture was subsequently used to inoculate 50 ml of DSM to an OD_600_ of 0.1. After the onset of sporulation, the OD_600_ of the culture was measured every hour for 6 h. At each time point, 1 ml of cells was collected and centrifuged at 6000 rpm for 6 min, and the harvested cell pellets were stored at −80 °C for later analysis. β-galactosidase activity was measured as described by Miller (46) with one modification. Prior to cell permeabilization, pellets were resuspended in 640 μl Z buffer (0.06 M Na_2_HPO_4_.7H_2_O, 0.04 M NaH_2_PO_4_.H_2_O, pH 7.0, 10 mM KCl, 1 mM MgSO_4_, 40 mM β-mercaptoethanol) and 160 μl of lysozyme (2.5 mg ml^−1^ in the Z buffer) was added. The suspension was incubated at 37 °C for 5 min. The subsequent steps were conducted according to the protocol described by Miller (46).

### Fluorescence microscopy and image acquisition

A single colony of each *B*. *subtilis* strain was resuspended in 100 μl of LB medium and spread onto LB plate. The lawn was grown overnight at 30 °C and subsequently used to inoculate 10 ml of DSM to an OD_600_ of 0.1. This culture was then grown at 37 °C and cells were harvested 2 and 3 h after the onset of stationary phase. For membrane visualization, the fluorescent dye FM 4 to 64 (Molecular Probes) was used at concentrations of 0.2 to 1 μg ml^-1^. Cells were examined under the microscope on 1% agarose covered slides. When it was necessary to increase the cell density, cells were concentrated by centrifugation (3 min at 2500 rpm) and resuspended in a small volume of supernatant before microscopic examination. All images were obtained with an Olympus BX63 microscope equipped with a sCMOS Zyla-4.2P camera (Andor). Olympus CellP imaging software (https://www.olympus-lifescience.com/en/software/cellsens) and ImageJ/Fiji (https://fiji.sc) were used for image acquisition and analysis.

## Data availability

Original microscopic images are available upon request. All data generated or analyzed during this study are included in this published article and its supporting information or available upon request. Source data are provided in this paper.

## Supporting information

This article contains supporting information (24, 35, 36, 43, 44, 45, 47, 48).

## Conflict of interest

The authors declare that they do not have any conflicts of interest with the content of this article.
