# Supplementary material for: Direct SpoIIQ-SpoIIIAH interaction is dispensable for sporulation in Bacillus subtilis
Source: J Biol Chem. 2025 Nov 12;301(12):110934. doi: 10.1016/j.jbc.2025.110934 (PMC12720343; doi:10.1016/j.jbc.2025.110934)
Supplement: Supporting Material [file mmc1.docx]

**Supporting Information**

**Direct SpoIIQ-SpoIIIAH interaction is dispensable for sporulation in *Bacillus subtilis***

Katarína Muchová, Andrea Vetráková, James A. Brannigan, Sonam Sidhu, Jana Júdová, Zuzana Chromiková, Anthony J. Wilkinson and Imrich Barák

**Table S1. Sporulation efficiency of *spoIIQ* mutant strains determined as heat resistance and reported as relative to wild type IB1856.**

| strain | relevant genotype | sporulation efficiency % |
| --- | --- | --- |
| IB1856 | *amy:: p_IIQ_-mgfp-spoIIQ spoIIQ::erm* | 100 |
| BKE36550 | *spoIIQ::erm* | 0.05±0.015 |
| IB1857 | *amy:: p_IIQ_-mgfp-spoIIQL109/118E spoIIQ::erm* | 79±8.9 |
| IB1858 | *amy:: p_IIQ_-mgfp-spoIIQL109A spoIIQ::erm* | 87±4.16 |
| IB1859 | *amy:: p_IIQ_-mgfp-spoIIQL109F spoIIQ::erm* | 92±6.43 |
| IB1860 | *amy:: p_IIQ_-mgfp-spoIIQL109E spoIIQ::erm* | 66±4.35 |
| IB1861 | *amy:: p_IIQ_-mgfp-spoIIQL118A spoIIQ::erm* | 17±4.40 |
| IB1862 | *amy:: p_IIQ_-mgfp-spoIIQL118F spoIIQ::erm* | 82±4.00 |
| IB1863 | *amy:: p_IIQ_-mgfp-spoIIQL118E spoIIQ::erm* | 93±7.50 |

**Table S2. Sporulation efficiency of *spoIIIAH-mscarlet* strains determined as heat resistance and reported as relative to wild type PY79.**

| strain | relevant genotype | sporulation efficiency % |
| --- | --- | --- |
| PY79 | wild type | 100 |
| KM1600 | *p_IIIAH_-spoIIIAH-mscarlet* | 96.3±4.66 |
| KM1601 | *p_IIIAH_-spoIIIAH-mscarlet amy::p_IIQ_-gfp-spoIIQ spoIIQ::erm* | 99±1.41 |
| KM1602 | *p_IIIAH_-spoIIIAH-mscarlet amy::p_IIQ_-gfp-spoIIQL109/118E spoIIQ::erm* | 84.5±2.12 |
| KM1603 | *p_IIIAH_-spoIIIAH-mscarlet spoIIQ::erm* | 0.04±0.025 |

**Table S3. Bacterial strains**

**Table S4. Plasmids**

**Table S5. Oligonucleotides used in this work**

**Figure S1**

**
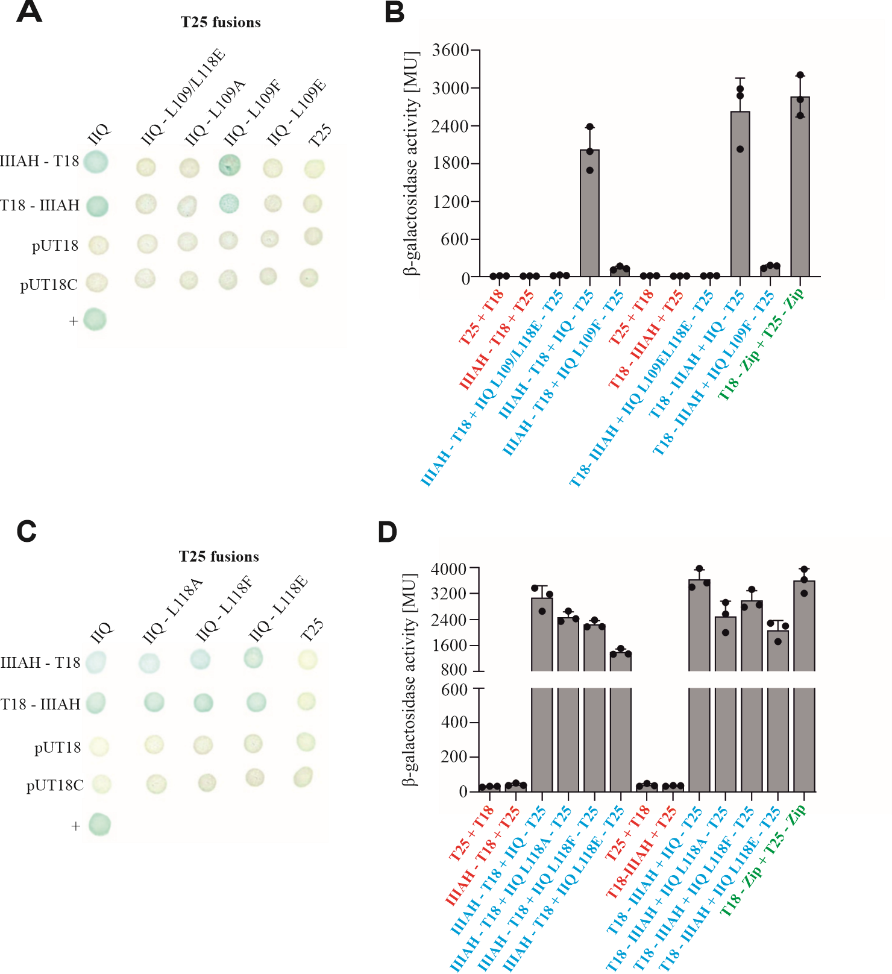
**

Figure S1. Interactions of extracytoplasmic part of SpoIIIAH with extracytoplasmic part of SpoIIQ variants. A. and C. BACTH showed different interaction properties of SpoIIQ variants. *E. coli* strain BTH101 (*Δcya*) was cotransformed with plasmids encoding the indicated fusions to adenylate cyclase fragments T18 and T25. Colonies were spotted on selective plates containing IPTG and X-Gal. The blue color indicates a positive interaction between each pair of fusion proteins. The positive control, T18-Zip+T25-Zip, is marked +. Fusions of extracytoplasmic part of SpoIIQ variants with fragment T18 are shown in main text. B. and D. BACTH β-galactosidase activity assays. Normalized β-galactosidase activity expressed in Miller units (MU) is shown. The mean values from each experiment were normalized to the negative-control (in red) values (BTH101 cells coexpressing only T18 and T25 subunits of adenylate cyclase). The values of specific negative controls (only one subunit of adenylate cyclase fused with the respective target; the second subunit remained free) are also shown. Each experiment was performed at least three times independently. The bars represent averages, and the dots represent individual experiments. Error bars represent ±SD. Red and green text in the x-axis description indicates negative (T25+T18, IIIAH-T18+T25) and positive controls (T18-Zip+ T25-Zip), respectively. Note that some negative interactions from A. and C. are not included in this assay. BACTH β-galactosidase activity assays of fusions of extracytoplasmic part of SpoIIQ variants with fragment T18 are shown in the main text.


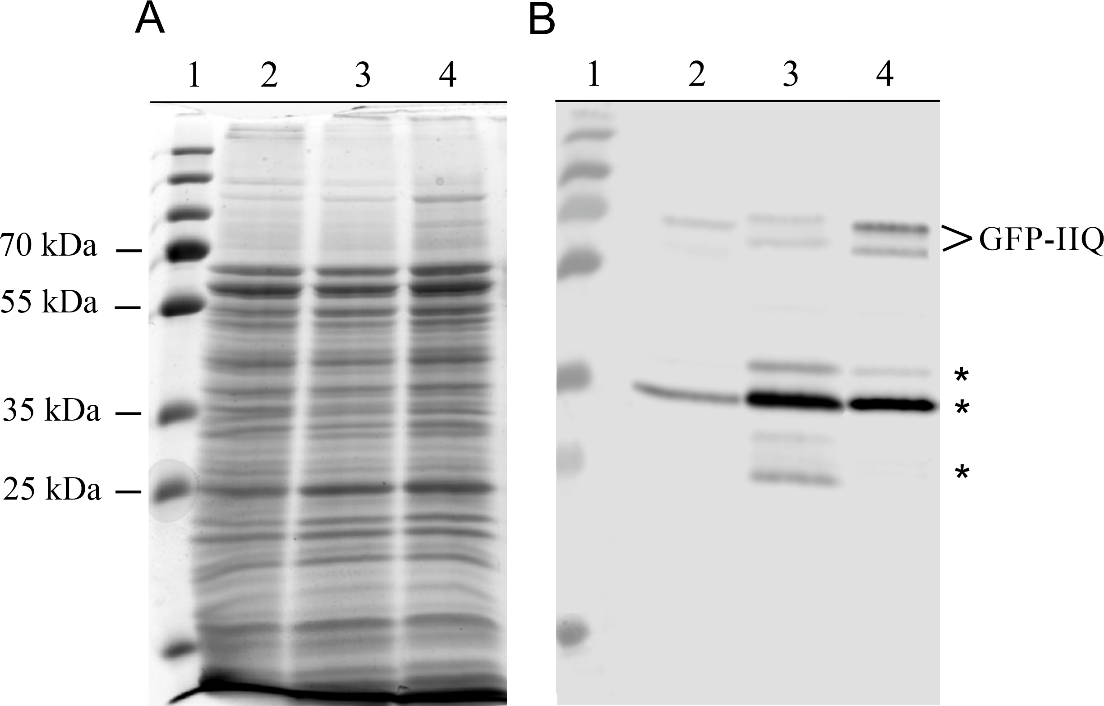


Figure S2. Western blot analysis of wild type GFP-SpoIIQ and the GFP-SpoIIQL109/118E mutant in *B. subtilis* strains. The cells were induced to sporulate and harvested at the 2nd hour of sporulation. Equal amounts of total protein from detergent-solubilized membrane fractions prepared as described in (36) were loaded for analysis. A. SDS-PAGE gel stained with Coomassie Brilliant Blue. Lane 1 – MW marker, 2 – GFP-SpoIIQ in strain IB1856, 3 – GFP- SpoIIQL109/118E in strain IB1857, 4 – GFP- SpoIIQL118E in strain IB1863. B. Western blot of the SDS-PAGE gel. Lanes are the same as in panel A. A monoclonal anti-GFP antibody (cat. number ab1218, Lot. GR213436-60, Abcam) was used for the detection of GFP-SpoIIQ. GFP-SpoIIQ variants (GFP-IIQ) migrate as the double bands ~68 kDa as was observed previously (35). The asterisks (*) indicate degradation products suggesting proteolysis during sample preparation as was also shown previously (35). This immunoblot shows that there are no large differences in the amount of full-length wild type SpoIIQ and the double SpoIIQ mutant (GFP-SpoIIQL109/118E).

**References**

24. Karimova, G., Pidoux, J., Ullmann, A., and Ladant, D. (1998) A bacterial two-hybrid system based on a reconstituted signal transduction pathway. *Proc. Natl. Acad. Sci. U. S. A.* **95**, 5752–5756

35. Jiang, X., Rubio, A., Chiba, S., and Pogliano, K. (2005) Engulfment-regulated proteolysis of SpollQ: Evidence that dual checkpoints control σ^K^ activity. *Mol. Microbiol.* **58**, 102–115

36. Campo, N., Marquis, K. A., and Rudner, D. Z. (2008) SpoIIQ anchors membrane proteins on both sides of the sporulation septum in Bacillus subtilis. *J. Biol. Chem.* **283**, 4975–4982

43. Lewis, P. J., and Marston, A. L. (1999) GFP vectors for controlled expression and dual labelling of protein fusions in Bacillus subtilis. *Gene*. **227**, 101–109

44. Guérout-Fleury, A. M., Frandsen, N., and Stragier, P. (1996) Plasmids for ectopic integration in Bacillus subtilis. *Gene*. **180**, 57–61

45. Youngman, P., Perkins, J. B., and Losick, R. (1984) Construction of a cloning site near one end of Tn917 into which foreign DNA may be inserted without affecting transposition in Bacillus subtilis or expression of the transposon-borne erm gene. *Plasmid*. **12**, 1–9

47. Koo, B. M., Kritikos, G., Farelli, J. D., Todor, H., Tong, K., Kimsey, H., Wapinski, I., Galardini, M., Cabal, A., Peters, J. M., Hachmann, A. B., Rudner, D. Z., Allen, K. N., Typas, A., and Gross, C. A. (2017) Construction and Analysis of Two Genome-Scale Deletion Libraries for Bacillus subtilis. *Cell Syst.* **4**, 291-305.e7

48. Backman, K., Ptashne, M., and Gilbert, W. (1976) Construction of plasmids carrying the cI gene of bacteriophage lambda. *Proc. Natl. Acad. Sci. U. S. A.* **73**, 4174–8
